# Two-dimensional photonic crystal with ring degeneracy and its topological protected edge states

**DOI:** 10.1038/s41598-019-40677-5

**Published:** 2019-03-07

**Authors:** Mengjia He, Li Zhang, Huaping Wang

**Affiliations:** 1 Key Laboratory of Micro-Nano Electronic Devices and Smart System of Zhejiang, College of Information Science and Electronic Engineering, Zhejiang University, Hangzhou, 310027 China; 2 Institute of Marine Electronics Engineering, Ocean College, Zhejiang University, Hangzhou, 310058 China

## Abstract

We propose a two-dimensional photonic crystal that possesses a degenerate ring in the momentum space. The photonic crystal is composed of the parallel-plate metal filled with a periodically arranged square array of metallic cylinders. Opening an air gap breaks the z-inversion symmetry, leading to the modes coupling (bi-anisotropy response) of TE and TM waves. This induced electric-magneto coupling, a similar role of the spin-orbit interaction in the condensed matters, results in a complete topological band gap around the degenerate frequency. The bulk bands below the band gap take non-zero *Z*_2_ topological invariant characterized by the evolution of the Berry phase. As a consequence, the interface of two photonic crystals with opposite bi-anisotropy supports topological protected edge states that exhibit one-way propagation and are highly resistant to disorders. Our work might be very useful for the design of topological photonic crystals and may serve as a platform for studying pseudo-spin photonics.

## Introduction

Recent discoveries in the field of topological non-trivial phases have led to a growing research in topological insulators and semimetals, and these concepts have been introduced to their electromagnetic counterpart communities. Various two dimensional (2D) photonic crystal systems have been proposed to emulate the quantum Hall effect^1,2^, quantum spin Hall effect^3–5^, and quantum valley Hall effect^6,7^, with particular interests in achieving one-way disorder immune edge waves. In three dimensional (3D) case, the existing literature mainly focus on 3D Dirac points^8,9^, Weyl degeneracy^10–14^, nodal line degeneracy^15,16^, and nodal chain^17^.

**Figure 1:**
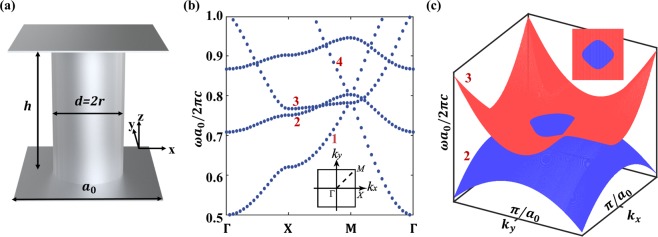
Schematic view of 2D photonic crystal with ring degeneracy. (**a**) A unit cell of the crystal, consisting of a metallic cylinder sandwiched in a parallel-plate metal $(h={a}_{0},r=0.276{a}_{0},{a}_{0}=5\,mm)$. (**b**) PBS of the 2D photonic crystal, where the second (TE) and third (TM) band crosses with each other around the M point. The insets show the corresponding first Brillion zone. (**c**) Three dimensional view of the PBS in the vicinity of M point,the amplified part exhibits a closed ring.

Among the 2D pseudo-spin or valley Hall photonic systems constructed by metallic^3,5,7,18^ and dielectric^6,19,20^ meta-atoms, the triangle lattice photonic crystals have been widely studied because they have the highest point group symmetry. The symmetry introduces special degeneracy in the momentum space and concomitant symmetry-protected topological photonic states, i.e., the topological protected edge waves. If the symmetry is removed, there are also some accidental degeneracies that are not guaranteed by symmetry^21^, it is of interest to study nontrivial topological phenomenon behind this accidental degeneracy. To discuss above situation, our start point is a 2D meta-waveguide structure that possesses an accidental degenerate ring in the momentum space, which differs from the four-degenerated Dirac points in a hexagonal array of metallic cylinders^5^. The ring degeneracy can possess non-trivial connectivity and consequent wave dynamics that are richer than point degeneracy^17^, i.e., degeneracy occurs in any propagation direction. For a square array of metallic rods, there are four corresponding bands correlating at the surrounding of one high-symmetry point in reciprocal space (see Fig. 1(b)). Specifically, one transverse electric (TE) mode and one transverse magnetic (TM) mode accidentally degenerate forming a closed ring. When breaking the z-inversion symmetry, the former bands interact with each other^22^ through magneto-electric coupling, resulting in a topological photonic crystal (TPC) with a complete band gap. To explicate the topological properties of the band gap, we calculate Z_2_ topological invariants characterized by the evolution of Berry phases. Such a TPC can support topological protected surface waves (TPSWs) characterized by its pseudo-spin state, which might be very useful to design robust integrated photonic components, such as sharp corner waveguides^23^, robust delay lines^6^, photonic networks^24^, and wave slitters^25,26^.

## Results

### Photonic band structure and topological Berry phase

The present photonic crystal with toroidal degeneracy is composed of two parallel-plate metal waveguides filled with a periodically arranged square lattice array of metallic cylinders. One unit cell is shown in Fig. 1(a), the radius of the cylinder *r* = 0.276*a*_0_ (*a*_0_ is the lattice constant). The metal is modeled as a perfect electric conductor, i.e., the tangential electric field vanishes at the metallic surface. Due to the z-inversion symmetry, the propagating waves in the meta-waveguide can be classified as either TE or TM waves. The TE/TM waves have non-vanishing electric/magnetic field out-of-plane components *E*_*z*_/*H*_*z*_, as well as the in-plane components ${H}_{\parallel }/{E}_{\parallel }$. The Eigen modes are calculated using the first-principles electromagnetic simulation software COMSOL Multiphysics, with periodic boundary along x, y direction and PEC boundary along z direction. Figure 1(b) shows the numerically calculated photonic band structure (PBS) of the meta-waveguide, the four-fold rotation symmetry ensures that the first and the third bands (both of them are TM modes) are quadratically degenerate at the M point^27^. By judiciously choosing *h* and the cylinders’ radius *r* under the condition of the given lattice constant, the second (TE) and third (TM) bands can touch with each other (accidental degeneracy). Here, the two modes strictly hold orthogonality and no coupling occurs, so the band structure will be the direct superimposition of the bands that belong to TE or TM modes. Figure 1(c) explicitly displays the dispersion relation in the vicinity of point M, where the amplified part exhibits a closed ring. That means two photonic bands intersect, forming a 2D closed degenerate ring in the momentum space.

**Figure 2:**
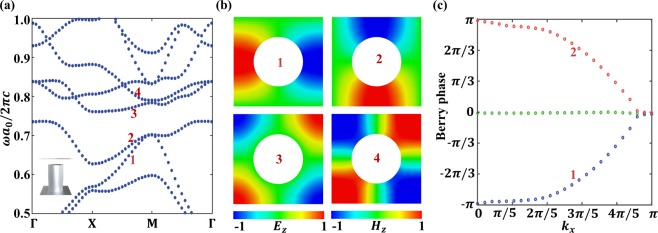
Band diagram and eigenmodes of the TPC and its topological phase. (**a**) PBS of the TPC. As the z-inversion symmetry is broken, the once degenerate TE and TM modes mix with each other, opening a complete band gap at the frequency range of 0.73 < *ωa*_0_/2*πc* < 0.76. The inset illustrates the unit cell, where the air gap perturbation (g = 0.1*a*_0_) causes bi-anisotropy response. (**b**) Fields profile of the four concerned modes at M point, out-of-plane magnetic component (*H*_*z*_) for first, second and fourth mode, out-of-plane electric component (*E*_*z*_) for third mode. (**c**) Berry phase of the bands below the band gap. It evolves with *k*_*x*_ ranges from 0 to π. Note that all the green dots reside in nearly zero phase, which corresponding to the lower band that is not explicated in (**a**).

Opening an air-gap in the meta-waveguide, as displayed in the inset of Fig. 2(a). This gap breaks the z-inversion symmetry and causes mutual coupling between the former orthogonal TE and TM modes, leading to the formation of new eigen values and the corresponding eigen states. This phenomenon also can be regarded as a bi-anisotropic response^28^, i.e., electromagnetic response of an optical medium with the bi-anisotropic tensor $\hat{\chi }$ is described by the following constitutive relations: $D=\hat{{\epsilon }}E+i\hat{\chi }H$and $B=\hat{\mu }E-i{\hat{\chi }}^{T}H$^29^. The effective electric-magnetic coupling term $\hat{\chi }$ can be calculated by perturbation theory and the values for the case that air gap residing in top and bottom of the waveguide is exactly opposite^5^. Due to the z-inversion symmetry breaking, the degenerate ring vanishes and a complete band gap forms in the frequency range of 0.73 < *ωa*_0_/2*πc* < 0.76, as shown in Fig. 2(a). Note that the band structure in Fig. 2(a) is identical for the meta-waveguide in which all the rods shift up or down, but these two configurations are topological distinct because they have opposite bi-anisotropy coefficient. The out-of-plane field patterns of the four bands in Fig. 2(b) indicate that the dipole modes are below the band gap and the quadrupole modes are above the band gap. In addition, the band structure at the M point are doubly degenerate for the dipole modes but not for the quadrupole modes, which distinguishes with the modes degeneracy in honeycomb lattice^30^.

Start from the accidental ring degeneracy, an introduction of air-gap perturbation breaks the z-inversion symmetry and causes evident band interaction (mode coupling), which leads to a nontrivial photonic topological band gap. To explicit the topology propriety of the band gap, we calculate the Berry phases^31,32^ of the two bands below the gap (see Fig. 2(c)), the blue and red dots correspond to the first band and the second band in Fig. 2(a), respectively. The multi-band non-Abelian Berry phases are calculated through the linking matrices $${M}_{mn}^{k,k+{\rm{\Delta }}k}$$ of the Bloch wave-functions ($${u}_{m,k})$$ between the two adjacent *k* points, i.e., $${M}_{mn}^{k,k+{\rm{\Delta }}k}=\langle {u}_{m,k}|{u}_{n,k+{\rm{\Delta }}k}\rangle $$, where the wave-functions can be obtained through Eigen mode simulation in COMSOL Multiphysics. When we fix *k*_*x*_, the Wilson loop reads

1 $${\rm{W}}({k}_{x})={\prod }^{}{M}^{({k}_{y},{k}_{y}+{\rm{\Delta }}{k}_{y})}$$

where the multiplication of the linking matrices along the closed loop, i.e., *k*_*y*_ ranges from 0 to 2π. The Berry phases come from the eigenvalues *λ*_*n*_(*k*_*x*_) of Wilson loop, i.e., ${\varphi }_{n}({k}_{x})=Im(\mathrm{log}\,{\lambda }_{n}({k}_{x}))$. With the Berry phase evolve when *k*_*x*_ ranges from 0 to π, we first draw an arbitrary reference line parallelling to the *k*_*x*_ axis, then compute the *Z*_2_ number by counting how many times the evolution line of the Berry phase crosses the reference line. For each band, its evolution line crosses the reference line only once, which determines the non-zero *Z*_2_ topological invariance. This non-zero *Z*_2_ number predicts the non-trivial topological edge waves residing in the boundary of the z-inversion symmetry breaking photonic crystal.

### Topologically protected edge states

**Figure 3:**
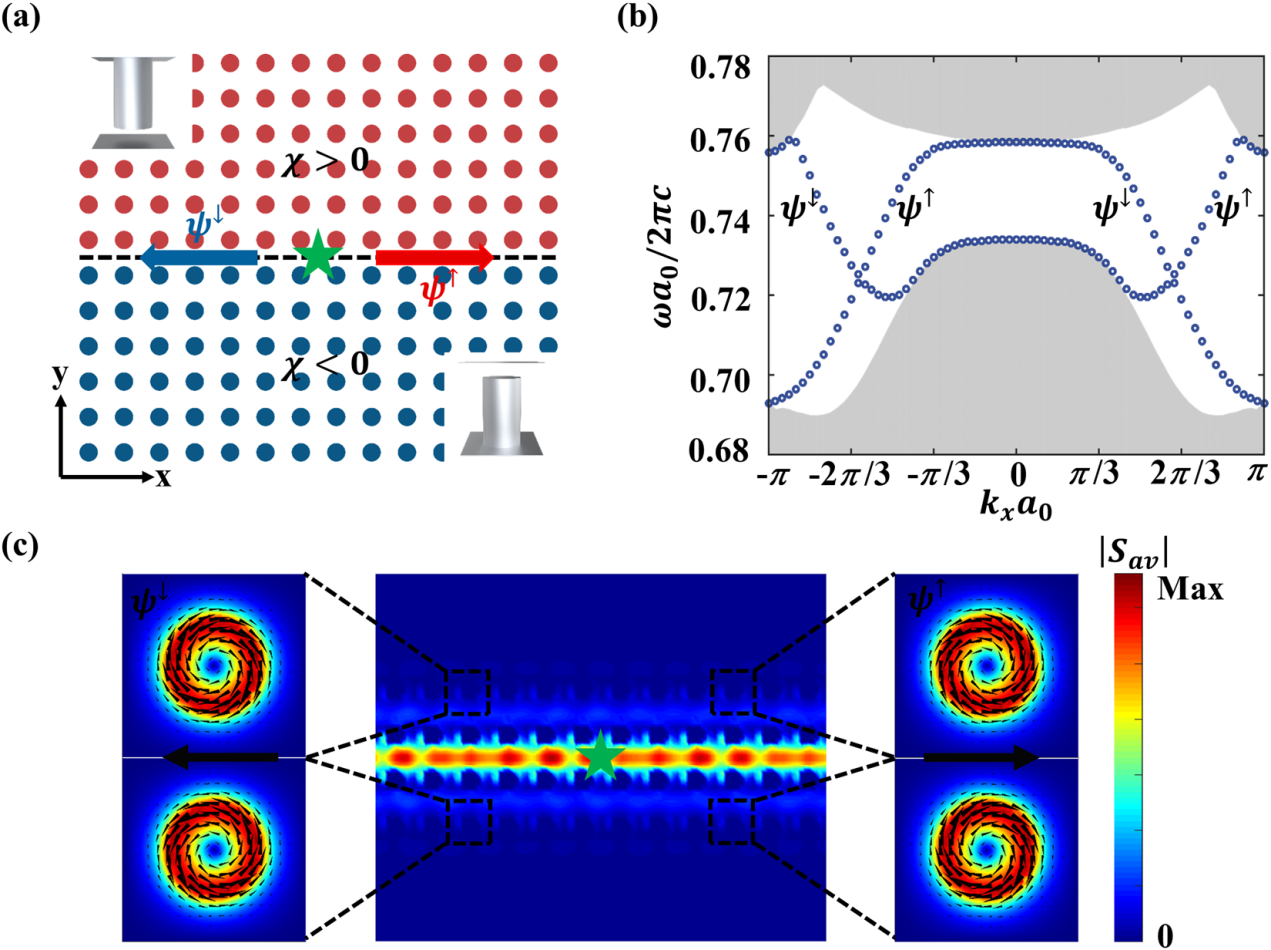
TPSWs at the interface between two TPCs with opposite bi-anisotropy. (**a**) Schematic view for a straight interface (dashed line) between two topological domains with cylinders shifted up (red color) and down (blue color). (**b**) One-dimensional PBS of a supercell (single cell along the x direction, 30 cells on each side of the interface) of the hybridized modes. Grey domain: bulk modes; blue and red lines: topological edge states. (**c**) Excitation of the edge states with a horizontal dipole (green star) at the normalized frequency (*ωa*_0_/2*πc* = 0.74). Color: energy flux density; arrows: Poynting vector. Spin locking ensures that the spin-up edge state propagate towards x direction while spin-down edge state towards the reverse direction.

Although the magneto-electric coupling opens a complete band gap and prevents photons from spreading into the bulk domain in specific wavelength range, the photonic wave can propagate along the interface in the form of edge states between two domains that have different topological invariants^18^. Considering an interface separating two locally homogeneous regions with opposite bi-anisotropy coefficients as shown in Fig. 3(a), the calculated band structure in Fig. 3(b) explicitly indicates that the interface supports two pairs of edge states, one forward-propagating pair and another backward-propagating pair, and each pair consists of two states with positive/negative refractive index. The field pattern in Fig. 3(c) is simulated with time domain solver in CST Microwave Studio; a vertical dipole (marked as green star) is placed at the middle of the interface to excite edge waves; the boundaries are open in the x-y plane and PEC in vertical direction. We can observe serval significant properties of TPSWs: first, the edge states indeed exist since their energy distribution is tightly confined to interface; second, the spatial polarization of topological edge states is closely related to the pseudo-spin state which directly decides the direction of propagation. The enlargement in Fig. 3(c) shows that pseudo-spin up edge state corresponds to right-hand/left-hand polarization of the power flux density in the upper/bottom meta-waveguide respectively while pseudo-spin down edge state is just the opposite. These vortex properties of the power flow in bulk area also demonstrate that the bi-anisotropic response leads to the so-called spin-orbit coupling of light^28^ which sustains the existence of topologically non-trivial edge states in optical spin Hall effect.

### Pseudo-spin locking in topological junctions

**Figure 4:**
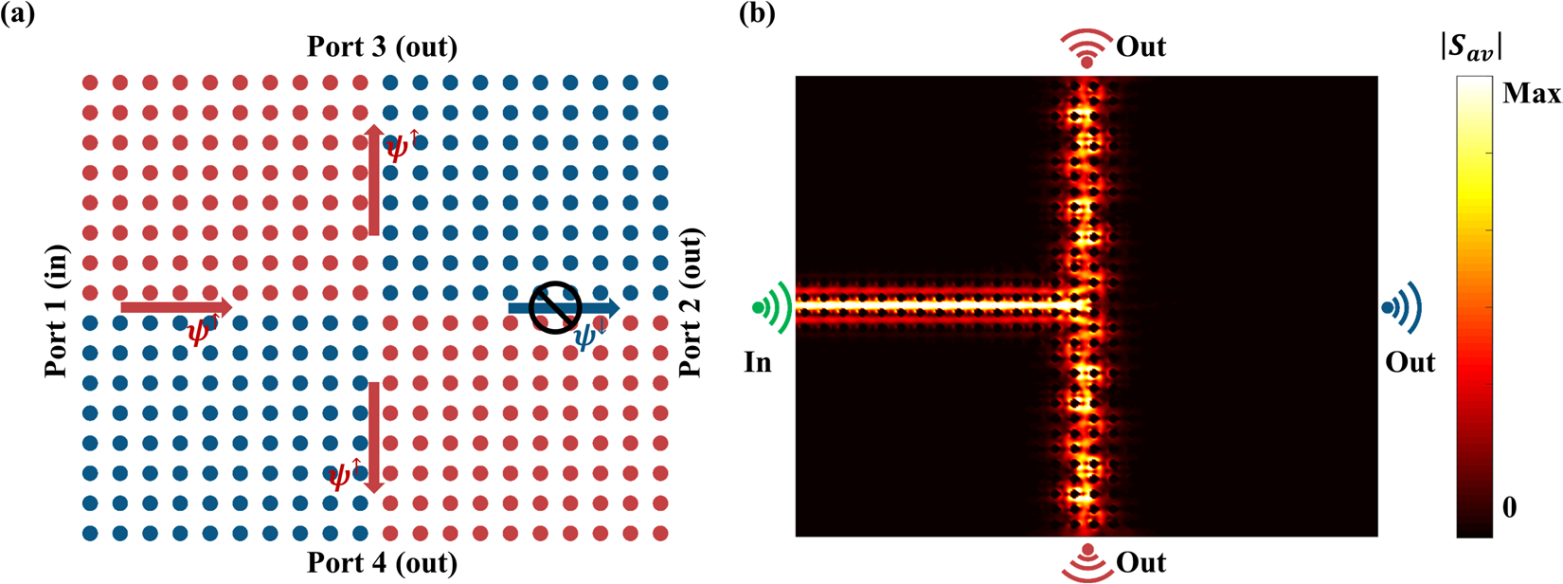
Spin-locking of an edge state at a four-port topological junction. (**a**) Schematic of a four-port topological junction constructed by four domains with rods shift up (red) or down (blue). A waveguide port excites a pseudo-spin up edge wave; spin-locking ensures that the edge wave can only couples into two of three domain walls and the transmission through port 2 is completed suppressed. (**b**) Numerical simulated energy flux intensity along the domain walls. The results agree well with above conclusion that the edge wave only travels along the top and bottom domain walls. Color scale: energy flux intensity at the normalized frequency (*ωa*_0_/2*πc* = 0.74).

To demonstrate that the pseudo-spin state directly determines the direction of propagation of the edge states, we consider two types of domain attribute to its bi-anisotropy coefficient, crossing in the center of the structure, as shown in Fig. 4(a). To elucidate the spin-locking propagation of the edge state, we intend to excite single edge state at the topological interface. Considering that the pseudo-spin is a superposition of basic waveguide modes, we use a waveguide port which excite TE and TM mode simultaneously with specific phase difference in the simulation. In Fig. 4(a), a waveguide port residing in port 1 excites a pseudo-spin up edge state, the left and right interfaces support it to travel towards the center while the top and bottom interfaces support it to travel away from the center. In the meanwhile, when a particular spin state arrives at the center, it cannot couple into another spin state because of the spin protected properties sustained by time-reversal symmetry. Hence the pseudo-spin up state excited by port 1 can only propagate through the top and bottom interfaces and be detected at port 3 and port 4. The energy flux shown in Fig. 4(b) reveals that the energy along the right interface has been completely suppressed for a pseudo-spin up state, which is consistent with above analysis. Note that the waveguide port can be replaced by an electrical dipole that can excite two edge states simultaneously while only one state can travel along the interface, i.e., if the dipole is placed at port 1, only the pseudo-spin up states can couple into left domain wall and propagate forward. In this sense, the junction can also be called a spin splitter which selects the spin up or down edge states.

### Robustness of edge states immune to disorders

**Figure 5:**
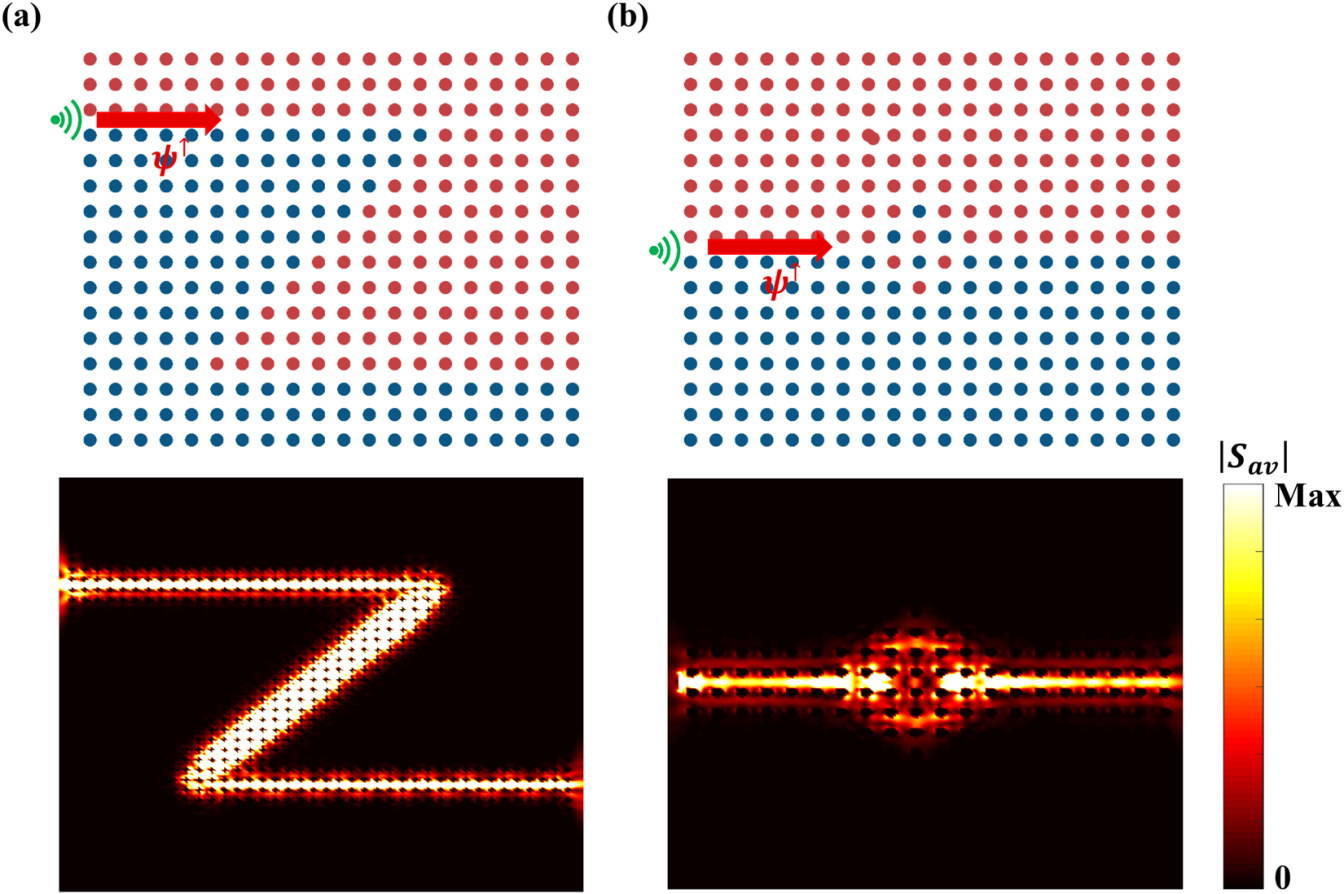
Robustness of TPSWs. (**a**,**b**) TPSWs along interfaces with different defects: sharp corners (**a**), and a strongly disordered domain wall (**b**). The top parts show the schematic structure: the red and blue area correspond to rods shift up and down respectively; the green marks indicate a waveguide port which excites a pseudo-spin plus edge state; the bottom parts are strength of the power flow. Color scale: energy flux intensity at the normalized frequency (*ωa*_0_/2*πc* = 0.74).

One of the most exotic properties of electronic topological insulators is the robustness of edge states to the time reversal (TR) symmetry preserved interfacial perturbation^4^. The same robustness exists in present TPC due to the spin-locking properties of the edge waves. It is well-known that TR symmetry guarantees pseudo-spin conservation, hence the topological photonic structure can support a topologically protected transport of edge waves: their reflection is strongly suppressed even in the presence of various structural perturbations^4,5,18^. Two types of the interface defects shown in Fig. 5(a,b) are investigated to demonstrate this property of edge waves. Note that both of these defects are non-magnetic that won’t break TR symmetry. In CST time domain solver, a waveguide port is placed at the entrance of the interface. To excite pseudo-spin plus edge state, two orthogonal modes with zero phase difference are excited at a normalized frequency $({\rm{\omega }}{a}_{0}/2\pi c=0.74)$, and the boundaries are the same as those in Fig. 3(c). The first type of interfacial perturbation shown in Fig. 5(a) includes two sharp bands along the interface between the two meta-waveguides with opposite bi-anisotropy. The power flow configurations (see Fig. 5(a)) conform to each segment of the interface, which indicates that the forward-propagating edge state transmits through the two sharp corners. The standing waves and localized modes cannot form along the propagation path as the edge state maintains spin degree and does not suffer back-reflection. Figure 5(b) shows a strong two-dimensional disorder perturbation which is formed by randomly shifting rods near the interfacial center, modelling a local fusing of the two meta-waveguides. It can be observed that although the energy spread into the disordered region, the backscattering is still suppressed, confirming that the edge states have manifested the topological robust to interface defects.

## Discussion

In this paper, we propose a 2D photonic crystal based on a meta-waveguide, which possesses a degenerate ring in the momentum space. By breaking z-inversion symmetry, the magneto-electric coupling (bi-anisotropic) arises between TE and TM modes near the ring, which mimics the spin-orbit coupling in electronic systems, leading to a complete topological band gap. The interfaces between two topological photonic crystals with opposite bi-anisotropy coefficient support TPSWs that has many significant characters. Such topologically extraordinary states can be described with a conserved pseudo-spin locked with the momentum. As confirmed by our theoretical analysis and simulations, the pseudo-spin locking property of TPSWs enables themselves robust to interface disturbances. Engineering a photonic meta-waveguide based on our design may provide a platform for reflection-free optical and microwave applications. Besides, our work might be very useful in the design of topological photonic crystals and may serve as a platform for studying pseudo-spin photonics.

## Data Availability

The data that support the plots within this paper and other findings of this study are available from the corresponding author upon reasonable request.
